# Book Reviews

**Published:** 1899-01

**Authors:** 


### Book Reviews.

A MANUAL OF SURGERY. For Students and Practitioners. By WILLIAM ROSE, M. B., B. S. Lond., F. R. C. S.; and ALBERT CARLESS, M. S., Lond., F. R. C. S. One volume, 8vo, of 1,162 pages, very profusely illustrated by wood engravings. New York: William Wood & Co. 1898.

This is by far the most pretentious manual in size pertaining to surgery that has been issued in many a year. An examination of its material, however, does not bring disappointment, for it everywhere betrays the work of careful hands—of skilful surgeons.

The authors declare it to be their purpose in creating the work and offering it to the profession, to present scientific facts in surgery in a concise and succinct form, especially to meet the necessities of the student, even when preparing for the higher examinations. They have also been constrained not to overlook the needs of the general practitioner of medicine, and on this account have undertaken the discussion in considerable detail those subjects he is most likely to meet in every-day practice. To meet both these conditions has not been an easy task, since there is such a wealth of material from which to choose, that the danger has been that the book world be overweighted, or exceed the limits of a manual. This, however, has been happily avoided by eliminating historical and bibliographical references, by excluding the consideration of special fields, such as the eye, ear and gynecological diseases, and by omitting some other questions frequently dealt with in similar works.

Due attention has been paid to the fact that surgical work has been transformed to a considerable extent by the progress of our knowledge in bacteriology and antisepsis. These questions have been duly considered where applicable throughout the book, which is in almost every respect a thoroughly modern treatise. The arrangement, too, is excellent. Inflammation is first considered and then its sequelæ; afterward new growths, injuries and diseases in their pathology, diagnosis an treatment, and, lastly, amputations and anesthesia. This is logical; some authors almost reverse the plan, or at least take up the last two subjects before they are reached in their proper order of sequence. The illustrations have been carefully chosen with a view to elucidate the text, but their number—392—might have been considerably increased without harm. Considered as a whole, however, this is one of the best of the later surgical manuals offered to students and practitioners.

A Text-Book of Practical Therapeutics: With Especial Reference to the Application of Remedial Measures to Disease and Their Employment upon a Rational Basis. By Hobart Amory Hare, M. D., Professor of Therapeutics and Materia Medica in the Jefferson Medical College of Philadelphia. With special chapters by Drs. G. E. de Schweinitz, Edward Martin and Barton C. Hirst. New (7th) edition. In one 8vo volume of 770 pages, illustrated. Philadelphia and New York: Lea Brothers & Co. 1898.

The regularity with which successive editions of this treatise appear—averaging once a year for seven years—entitles it to be classed as an annual. This fact speaks louder than can any words of the reviewer in its favor and must be most gratifying to both author and publisher. To prepare the copy for the press of such a comprehensive work every year, especially where careful revision is made and improvements added, entails an enormous amount of labor. Add to this all the other literary work of the author, besides the demands of a busy practice, and we may well wonder where all the time, to say nothing of the strength needed, comes from. There must be an enormous storehouse filled with these important elements at Dr. Hare's command from which he is permitted to draw *ad libitum*. We wish he would give us the secret key.

We have spoken so often in regard to this work, sometimes in considerable detail, especially regarding its early editions, that there remains little to add now, beyond the general fact, hinted at above, that careful revision and discreet additions have kept this text-book abreast of time and science in their onward march—this remark applying with cogency to this particular edition. This issue, too has been made to conform to the British pharmacopeia of 1898, as it always has done and continues to do to that of the United States.

Finally, we desire to invite particular attention to the indexes. First comes the index of drugs that fills sixteen double column pages; then follows an index of disease and remedies, occupying forty-three pages, also double columned. The latter is filled with full therapeutic hints and directions for treatment, with references to the text for fuller details. Nowhere in the literature of medicine have we seen such completeness of indexing, which contributes not a little to make this one of the best books of its kind extant. If a physician or student must limit himself to one work on therapeutics and our advice were sought, we should unhesitatingly say get Hare's.

---

An American Text-Book of Gynecology, Medical and Surgical. Edited by J. M. Baldy, M. D., Professor of Gynecology in the Philadelphia Polyclinic, etc. Second edition, thoroughly revised. Handsome imperial octavo volume of 718 pages, with 341 illustrations in the text and thirty-eight colored and half-tone plates. Philadelphia: W. B. Saunders. 1898.

It is less than five years since we were called upon to notice the first edition of this comprehensive treatise that forms one of a series put forth by this publisher and called American text-books. Yet it must be admitted that in these four or five years past there has been much improvement in methods, though we doubt if anything very new or original has been added to our stock of knowledge in the gynecological field. If we try and perfect that which we have on hand, so as to make it more useful in saving life or alleviating and preventing suffering we shall employ our time to its greatest advantage for woman kind.

In our review of the first edition of this text-book, published in Volume XXXIII., p. 559, *et seq.*, we went over it in detail, giving description and criticism, as is usual when a new candidate for favor appears. Now we shall only note the changes made, and refer our readers to our former notice for a more complete setting forth of the special features of the treatise, and its advantages as a work of reference.

To begin with we may mention that the editor has replaced more than forty of the old illustrations by new ones, that are intended to give a clearer understanding of methods. He prides himself that he does not illustrate specimens, and we concede that these are more proper in a medical journal or a society transaction volume than in a text-book. But while disclaiming to picture specimens, he yet proceeds to do so in a number of instances throughout the volume. In this edition the description of the preparation for each operation and the after-care of patients have been carried over to the chapters on technique and after-treatment—which, though enlarging these latter chapters, has relieved the body of the book by that much, and as also contributed to the avoidance of repetition. The chapter on bladder, urethra and ureters has been considerably changed, while the section on plastic work has been made over and greatly improved. This is very important since the profession is beginning to recognise that here is the real field for much important operative gynecology.

The section on hysterectomy, that has been rewritten and more elaborately illustrated, is to be especially commended. This is the most important operation in the whole range of surgery, when viewed as to its possibilities, and its results in saving life, or restoring women to health and happiness, absolute or relative, hence everything that will throw light on the subject or tend to simplify or lesson its dangers, is to be welcomed. This author cannot be to warmly praised for his useful work in this direction and for the intelligent setting forth he has given it in this book.

As a final remark we may be permitted to add that this text-book is to be regarded as a decided advance in the methods of teaching gynecology, and the present edition is to be commended for the way in which it has kept pace with the hurry that is everywhere attending the progress of events.

---

Pathology and Morbid Anatomy. By T. Henry Green, M. D., Lecturer on Pathology and Morbid Anatomy at Charing-Cross Hospital Medical School, London. New (eighth) American from the eighth and revised English edition. In royal octavo volume of 600 pages, with 215 engravings and a colored plate. Philadelphia and New York: Lea Brothers & Co. 1898.

We have heretofore had the pleasure of presenting to our readers notices of former editions of this excellent work, and we rejoice to be able to call attention to the new and improved issue of a standard authority on the subjects which it sets forth with so much clearness and accuracy. In the Journal for November, 1889, and again in the issue of March, 1896, we have discussed the value of this work with considerable detail, especially in the first named reference. Now we shall feel called upon to merely set forth some of the improvements which this edition contains.

In comparing this with former editions it will be discovered that a change has been made in the sequence of the text. The reviser, Dr. Walton Martin, of the College of Physicians and Surgeons, says this became necessary in order to give consideration not only to general pathologic laws, but to their application to special tissues and organs—a statement with which we quite agree. Some new paragraphs have been added in order to make the text read consecutively as well as smoothly. Some of the material of former editions has been omitted, an in its place is found a chapter on animal parasites; a new classification of tumors has been introduced, more in keeping with present methods of teaching; and, lastly, we may observe that a number of new illustrations have been introduced, while some less satisfactory in character have been omitted. This work is one of great importance to the student of pathology and morbid anatomy, for it is constructed on lines abreast of the present status of this branch of medical science; and, moreover, is one of the best treatises on the subject that has been published. The frequent demand for new editions attest the truth of this assertion and the further fact that it is quite generally used in the schools as a text, but accentuates it in a forceful manner. It is a book that no teacher of pathology can dispense with.

---

INTERNATIONAL CLINICS. A Quarterly of Clinical Lectures and Specially Prepared Articles on Treatment and Drugs. By professors and lecturers in the leading medical colleges of the United States, Germany, Austria, France, Great Britain and Canada. Edited by JUDSON DALAND, M D., (Univ. of Penna.), Philadelphia, Instructor in Clinical Medicine and Lecturer on Physical Diagnosis in the University of Pennsylvania; Assistant Physician to the Hospital of the University of Pennsylvania, etc.; Fellow of the College of Physicians of Philadelphia. J. Mitchell Bruce, M. D., F. R. C. P., London, England, Physician to and Lecturer on the Principles and Practice of Medicine at the Charing Cross Hospital. David W. Finley, M. D., F. R. C. P., Aberdeen, Scotland, Professor of Practice of Medicine in the University of Aberdeen; Physician to and Lecturer on Clinical Medicine in the Aberdeen Royal Infirmary, etc. Volume III. Eighth Series. 1898. Octavo, pp. xii.—355 Philadelphia: J. B. Lippincott Co. 1898.

Among the eminent American physicians and surgeons who contribute to the interest to this volume we may mention James M. Anders, of Philadelphia; J. Henry Carstens, of Detroit; John B. Hamilton, of Chicago; Joseph M. Mathews, Thomas Hunt Stucky, of Louisville, and Paul F. Mundé, of New York. In the foreign group we observe the names of T. Lauder Brunton, Sir Dyce Duckworth, Stull Graham, Frederick Trendelenburg, and Anton Wölfler. To these, with others, many of whose names are familiar in the literature of medicine, but which must be omitted for lack of space, we are indebted for one of the most interesting books in the series. In Charles Warrenne Allen's lecture on the treatment of certain skin affections there is a most excellent photographic reproduction of epithelioma of the nose, both before and after treatment. There are a number of plates and drawings, illustrating the text—a few more than some of the previous volumes have contained. This improvement is to be encouraged, for nothing serves to give zest to a printed clinical lecture so much as a proper illustration of the subject of the discourse.

A TEXT-BOOK OF MATERIA MEDICA, THERAPEUTICS AND PHARMACOLOGY. By GEORGE F. BUTLER, Ph. G., M. D., Professor of Materia Medica and of Clinical Medicine in the College of Physicians and Surgeons, Chicago. Second edition, thoroughly revised. Handsome octavo volume of 860 pages, illustrated. Philadelphia: W. B. Saunders. 1898.

A second edition of this book demanded and issued less than two years after the first speaks well for its reception. In our notice of the work when it first appeared (see the JOURNAL, February, 1897,) we pointed out a few of its excellent qualities and predicted its success. Since then there have been many improvements in pharmacology, chiefly in the direction, as the author states, in clearing from obscurity the material already at hand, rather than in recording recent discoveries of a startling nature.

The physiological action of drugs is constantly receiving more and more attention, and some additions have of late been made to our knowledge of this subject as relates to antipyretics, antiseptics, aconite, strychnine and a few others, all of which are here recorded. A chapter on the untoward effects of drugs has been substituted for that on definitions, while serum-therapy and the therapeutics of nuclein have been fully discussed in this edition.

The arrangement of the book is admirable, its type is clear, and its pages are most pleasing to the eye. This, in short, is one of the books that adds to the completeness of a physician's or a pharmacist's library and we feel sure that it will find its way into many of them—indeed all of the best.

Elements of Histology. By E. Klein, M. D., F. R. S., Lecturer on General Anatomy and Physiology in the Medical School of St. Bartholomew's Hospital, London; and J. S. Edkins, M. A., M. B., Joint Lecturer on and Demonstrator of Physiology in the Medical School of St. Bartholomew's Hospital, London. In one 12mo volume of 506 pages, with 296 illustrations. Philadelphia and New York: Lea Brothers & Co. 1898.

A knowledge of the intimate structure of the tissues of the body is fundamental so that their study should begin with that of anatomy itself. This manual on the subject, now in its fifth edition, is one of the best of its kind. Its teachings are clear, concise, and at the same time scientific. Moreover, its size and form make it most convenient for the student.

In this edition a thorough revision of the text has been made, and about 125 pages, as well as over 100 engravings have been added, thus bringing it forward to the present time in its teachings. The recent progress in the knowledge of cell-life, and the discoveries in the structure of the central nervous system, as well as organs of sense, are herein set forth with remarkable precision and conciseness. It is certainly one of the very best elementary treatises with which we are familiar, having been adopted by most of the colleges as a working guide for students, and besides it contains all that is requisite for the general practitioner of medicine.

American Pocket Dictionary. Edited by W. A. Newman Dorland, A. M., M. D., Assistant Obstetrician to the Hospital of the University of Pennsylvania; Fellow of the American Academy of Medicine, etc., etc. Containing the pronunciation of over 26,000 words, along with over 60 extensive tables. Philadelphia: W. B. Saunders, 925 Walnut Street.

Every medical student needs a good condensed dictionary—usually called pocket dictionary—that can be easily carried, and that is available at all times for immediate reference. This book is emphatically such an one and gives pronunciation as well as definition—an important consideration. Its price, $1.25, brings it within the range of the limited purse, which is another feature that adds to its usefulness It readily takes a front rank with books of its class.

THE MEDICAL RECORD VISITING LIST, OR PHYSICIAN'S DIARY FOR 1898. New revised edition. New York: William Wood & Co. 1899.

This is one of the handsomest and most practical of visiting lists. It contains calendars, tables, emergency hints, beside space for daily record of patients, obstetric engagements, consultation practice, cash accounts, special memoranda, etc., etc.. It, indeed, embraces everything the busy physician needs in such a book; besides, it is well printed and substantially as well as beautifully bound in seal leather, with broad flap and pencil holder, which contains a good pencil. This diary is too well known to need extended description or fulsome praise at our hands. No mistake can be made in choosing this book, as it is most satisfying in all respects.

---

THE ESSENTIALS OF HISTOLOGY. By EDWARD A SCHAFER, F. R. S., Professor of Physiology in University College, London. New (5th) edition. Revised and enlarged. Octavo, 350 pages, with 325 illustrations. Philadelphia and New York: Lea Brothers & Co., Publishers.

A new edition of this excellent treatise, which has steadily advanced to the dignity of a text-book will be welcomed by all students of structural anatomy. In this revision fifty pages of material and sixty-seven engravings have been added. The illustrations are especially clear and are very helpful to the beginner. The author makes his description simple and easily comprehended, while at the same time this book is valuable to the advanced student and teacher. His directions, too, for the microscopical examination of tissues are simple and clear. It is a book in which the essential facts pertaining to histology are delineated.

